# Emerging Approaches for Validating and Managing Multiple Sclerosis Relapse

**DOI:** 10.3389/fneur.2017.00116

**Published:** 2017-03-29

**Authors:** Elizabeth A. Mills, Ali Mirza, Yang Mao-Draayer

**Affiliations:** ^1^Molecular and Behavioral Neuroscience Institute, University of Michigan Medical School, Ann Arbor, MI, USA; ^2^Graduate Program in Immunology, Program in Biomedical Sciences, University of Michigan Medical School, Ann Arbor, MI, USA; ^3^Department of Neurology, University of Michigan Medical School, Ann Arbor, MI, USA

**Keywords:** pseudo-relapse, cortical lesion, biomarker, comorbidity, estrogen

## Abstract

The autoimmune disease multiple sclerosis (MS) is characterized by relapses in the majority of patients. A definitive clinical diagnosis of relapse in MS can be complicated by the presence of an infection or comorbid disorder. In this mini-review, we describe efforts to develop enhanced imaging techniques and biomarker detection as future tools for relapse validation. There is emerging evidence of roles for meningeal inflammation, sex hormones, comorbid metabolic or mood disorders, and a dysregulated immune profile in the manifestation and severity of relapse. Specific subsets of immune cells likely drive the pathophysiology of relapse, and identification of a patient’s unique immunological signature of relapse may help guide future diagnosis and treatment. Finally, these studies highlight the diversity in terms of relapse presentation, immunological signature, and response in patients with MS, indicating that going forward the best approach to assessment and treatment of relapse will be multifactorial and highly personalized.

Multiple sclerosis (MS) is a chronic autoimmune disease, which most commonly presents in a relapse and remitting form, called relapsing-remitting MS (RRMS). Approximately 85 to 90% of patients with MS will experience one or more relapses, also called flares or exacerbations, at some point in the course of their disease (1). MS relapses can manifest as a wide array of symptoms, including fatigue, sensory disturbances, and ataxia. Relapse severity generally increases, while recovery decreases with age and disease progression (2). Since relapses are associated with inflammatory demyelinating lesions, research in recent years has been devoted to uncovering the role of the immune system and other physiological factors involved in triggering relapse in RRMS. Here, we describe recent advances regarding the etiology, characterization, and validation of relapse.

## Determining the Validity and Severity of a Relapse

Relapses in RRMS are related to increased inflammatory activity associated with central nervous system (CNS) demyelination and new or worsening symptoms lasting at least 1–3 days, but can persist for months. The location of the demyelinating lesion affects the presentation of symptoms for a given relapse. Due to the etiology of a relapse, it is possible for patients to experience a return of symptoms when the body enters a pro-inflammatory state unrelated to their autoimmune disorder, such as in the context of an infection. In these instances, the patient experiences a “pseudo-relapse.”

A pseudo-relapse can also be associated with non-inflammation-related changes in body temperature, which can occur during fever, heat exposure, or exercise. In patients with demyelinated lesions, increases in body temperature can impact the ability of an action potential to propagate along an axon, resulting in a conduction block (3). Under these conditions, then, a patient will typically experience a return or worsening of previous symptoms. The physical exam is the primary method used to diagnose relapse, while magnetic resonance imaging (MRI) is often used to help validate the authenticity of a relapse. Gadolinium enhancement of T1-weighted MRI can detect white matter lesions indicative of breakdown of the blood–brain barrier. The finding of a new lesion indicates recent demyelinating activity, and if anatomically consistent with the nature of the symptoms, supports the diagnosis of a true relapse. It is important to be able to differentiate relapse from infection or other medical comorbidities and treat the underlying illness first.

**Figure 1:**
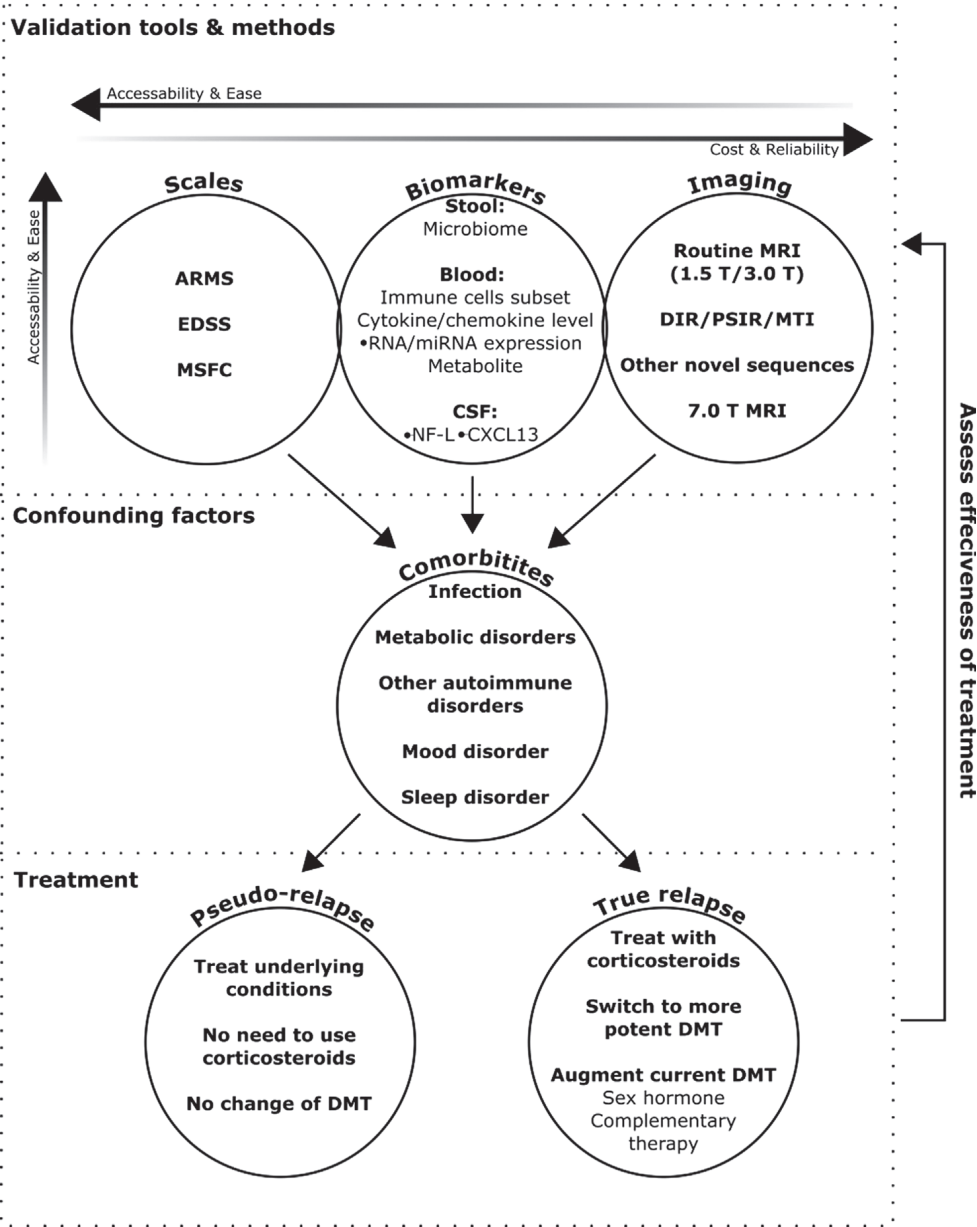
**Emerging methods to validate and monitor treatment for multiple sclerosis (MS) relapse**. This schematic provides an outline of how currently available and emerging tools could be used to help validate the diagnosis of MS relapse, as determined through a physical exam. Assessment scales are currently the easiest, but subject to patient biases, and advanced imaging techniques the most reliable, but least accessible. Biomarkers are emerging as a new avenue to bridge this divide, but still require further clinical validation. Diagnosis can be confounded by comorbid disorders, the presence of which can also impact treatment decisions. If patients are diagnosed with a true relapse, the choice of treatment will depend on severity, and effectiveness can be monitored using the relapse validation tools.

The combination of patient symptoms, objective physical exam findings, and neuroimaging results should be compared to previous exams to help determine whether a patient is truly having a MS relapse. This comparative approach would be greatly facilitated by the increased use of existing symptom and disability assessment scales and the validation of new ones. The Expanded Disability Status Scale (4) is the most widely used neurological exam scale in clinical trial settings, but focuses primarily on mobility, while the Multiple Sclerosis Functional Composite (5) scale is considered more sensitive (6), however, both are time consuming. In contrast, the assessing relapse in multiple sclerosis (ARMS) questionnaire (7) is a less rigorous, but more practical method. If formally validated, the ARMS questionnaire could allow clinicians to more effectively evaluate a patient’s perceptions of relapse symptoms and quickly identify issues associated with a true or pseudo-relapse. Although better methods are needed to accurately assess the validity and severity of relapse in routine clinical practice, new tools have emerged in recent years to facilitate future diagnosis and treatment decisions (Figure 1) with the goal of improving patient outcomes.

## Cortical Pathology in Relapse

Cortical pathology and cognitive dysfunction are typically associated with progressive forms of late stage MS; however, recent studies suggest that gray matter damage begins earlier and can increase the severity and progression of RRMS (8). Historically, cortical demyelinating lesions could not be detected using conventional MRI techniques. These MRI “invisible” lesions could only be accurately identified through a pathological assessment using cortical tissue obtained through autopsy or biopsy, which made it impractical as a useful clinical diagnostic tool. Although more common in secondary progressive MS (SPMS), pathological evidence of cortical lesions has also been found in tissue obtained from patients recently diagnosed with RRMS (9).

Subpial cortical lesions are often associated with nearby meningeal inflammation. Tertiary lymphoid follicles, which are ectopic CNS lymphoid structures that attract and maintain B cells and T cells (10), have been found to reside in the meninges of some MS patients (11). Since the subarachnoid space contains the interface between cerebrospinal fluid (CSF) and vasculature, it can serve as an entry point for antigen-experienced leukocytes into the CNS. Indeed, in the rodent experimental autoimmune encephalomyelitis (EAE) model, B cell accumulation in the subarachnoid space and meningeal inflammation is associated with clinical symptoms of relapse (12). Although it has not yet been technically possible to perform a corollary analysis of this type in humans, pathological studies have indicated that the presence of these tertiary lymphoid follicles is associated with an increased severity of disease progression (11). Therefore, patients with and without cortical lesions or ectopic follicular structures may represent subpopulations with different disease trajectories, which could influence treatment options (8), and necessitates the development of better detection methods for these features.

Recent developments in MR imaging techniques have allowed for the detection of cortical lesions *in vivo* and provide for the chance to correlate imaging and pathological findings. The use of these more sensitive methods including magnetization transfer imaging, double inversion recovery, and phase-sensitive inversion recovery MR sequences improves detection of gray matter lesions, particularly when done in a high field 7-T scanner (13). These imaging techniques have provided evidence that clinical symptoms can be associated with cortical lesions during relapse (14). Advanced imaging techniques have also allowed for the detection of leptomeningeal inflammation, which in some cases has been pathologically confirmed to be both associated with follicular structures and in close proximity to subpial demyelinating lesions (15). Similar to prior pathology-based studies, leptomeningeal enhancements were associated with increased disability, and although more common in progressive patients, they were also found in 19% of examined RRMS patients. Interestingly, this study hints that, in RRMS patients, inflammation in the meninges may be transiently associated with cortical lesions and responsive to treatments during relapse. These new techniques will help improve our understanding of how cortical lesions occur in RRMS patients and the ability to do longitudinal studies assessing the effect of cortical lesions on relapse severity and progression from RRMS to SPMS.

## Biomarkers for MS Relapse

The detection of gadolinium-enhanced T1 lesions is currently the “gold standard” in terms of authenticating relapse in RRMS. However, MRI is a costly and time-intensive procedure and not the most practical diagnostic tool, particularly for patients who reside far from major medical centers. Additionally, the frequent use of gadolinium-based contrast agents can result in the development of gadolinium brain deposits in some patients (16). Unfortunately, an alternative imaging technique, diffusion-weighted imaging suffers from a high false positive rate (17), and traditional MS diagnostic markers in CSF, such as IgG, and oligoclonal bands, have not been shown to carry prognostic value for relapse (18). Since biomarkers have been used to identify and predict relapse in rheumatoid arthritis (RA) (19), research in recent years has sought to find a similar panel of markers for RRMS. At this point, no definitive markers have been identified, but some promising candidates have emerged.

RRMS is associated with demyelinating inflammatory activity and the immune cells driving this inflammation have particular biochemical signatures in terms of the genes they express and substances they secrete. Expression studies in MS patients have revealed major changes in immune system-related genes that are associated with the presence or severity of disease, particularly in B cells and T cells (20). Consequently, there has been a recent focus on the identification of factors, which convey the state of the immune system, in addition to those associated with active demyelination, in order to uncover a signature for MS relapse.

As a disease of the CNS, the most accurate indicators of RRMS disease activity are likely to reside within the CNS. Therefore, CSF potentially represents the best source for biomarkers. Levels of neurofilament light (NF-L) in CSF from MS patients has been found to be increased during active relapse and correlated with disease activity (21); however, NF-L is associated with axon loss in a variety of neurodegenerative disorders (22), and thus is not truly specific to RRMS relapse. The chemokine CXCL13 is indicative of inflammation and has also been found to be elevated in the CSF during relapse (23). As a potent B cell attractant involved in lymphoid follicle formation, we believe it may also serve as a readout of meningeal inflammation and CNS tertiary lymphoid structures, though that has yet to be tested. Similar to NF-L, increased CXCL13 levels are not specific to MS, but, in combination, may be more diagnostically predictive. Moreover, although CSF markers may be the most direct readouts of disease activity, obtaining CSF samples through lumbar puncture to validate relapse may not be clinically practical, which has propelled the effort to identify serum biomarkers.

The prognostic utility of peripheral serum biomarkers is supported by the diagnostic reproducibility of factors contained in both CSF and blood. Indeed, serum levels of both NF-L (24) and CXCL13 (25) are also associated with RRMS disease activity. mRNA expression studies in peripheral blood mononuclear cells (PBMCs) have shown that expression of the T cell cycle regulator RGC-32 decreases, whereas the cytokine IL-21 increases during acute relapses, compared to patients with stable RRMS (26).

Small non-coding RNAs, particularly microRNAs (miRNAs), which can be secreted in extracellular vesicles, are critical for the development and regulation of immune cells (27). Thus, assaying miRNAs from PBMCs and sera indicate not only the overall inflammatory state but also specify whether the immune system is acting in a beneficial or pathogenic manner. miR-155, which promotes pathogenic helper T cell and pro-inflammatory myeloid cell activation, is elevated during relapse and responds to disease-modifying therapy (DMT) (28).

Another study found evidence of small non-coding RNAs, which change in a bidirectional manner between relapse and remitting phases (29), including miR-18b, which has also been associated with poor prognosis in B cell lymphoma (30). Interestingly, some of these were sex dependent (29) and may be related to the role of estrogen in immune system regulation. Since infections can trigger similar immune responses, the use of immune system-associated factors alone would not be able to definitely discriminate between a true relapse and a pseudo-relapse. Once again, reliability would be increased by the incorporation of demyelination-related markers.

Stool samples, as a readout of the gut collective microbial community (microbiota), may also be the valuable source of biomarkers for MS relapse [for comprehensive review of the topic, see Ref. (31)]. The microbiota is a key integrated component of human biological systems, which influences the physiological systems of the host, particularly the immune system, both systemically (32) and in the CNS (33). The intestinal microbial community can regulate blood–brain barrier permeability (34) and demyelination (35) and thus may be associated with MS relapse risk (36, 37). Indeed, a recent pilot longitudinal study in pediatric MS patients found that the absence of the phylum Fusobacteria in the gut was associated with an increased risk of relapse compared to patients in which this phylum was present (38). Further studies will be required to confirm these findings and determine if they are predictive of relapse.

A recent study exploring the use of serum-derived cytokines as biomarkers in pediatric MS demonstrating that the ratio of IL-10 to other pro- and anti-inflammatory cytokines has predicative power for relapse (39) highlights the need for a comparative approach involving several markers. Additionally, since treatments affect many of these markers (40), it will be critical to have a baseline reading for each patient, and then a longitudinal assessment following treatment, looking at the profile for both relapsing and remitting conditions to determine which markers have the greatest prognostic value for a particular patient. Furthermore, the concentration of some inflammation-related serum biomarkers varies in a circadian manner, thus taking into account the time of day that samples are collected is vital to make meaningful comparisons (41). It is also possible that the validity of particular markers will change over the course of disease progression. Ultimately, the use of biomarkers is going to need to be multifactorial and highly personalized.

## Comorbidities

Comorbidities have been shown to affect disease progression, time to initiation of DMT, as well as compliance (42), which may be related to the increased mortality of these patients as compared to the general MS population (43). The presence of a comorbidity can affect the onset and severity of relapse. Comorbidities can negatively impact sleep in MS patients (44), which can, in turn, lead to a worsening of symptoms, especially fatigue and pain. Sleep is critical for the proper functioning of the immune system. The circadian regulation of cytokine output produces a daily rhythm in the inflammatory profile, with a pro-inflammatory state occurring at night (45). Disrupted sleep can interfere with this pattern leading to prolonged periods of inflammation throughout the day, thereby exacerbating symptoms (46). Additionally, the circadian rhythmicity of key components of the immune system has been shown to be dysregulated in MS patients (41). Circadian sleep disorders are common in MS patients (47) and could be tied to a disruption in melatonin production, which is important in sleep–wake cycle regulation. Melatonin helps dampen the overactive immune system (48) and low levels are associated with relapse (49) and depression (50). Moreover, sleep disturbances and fatigue are strongly associated with depression in MS patients (51).

Psychiatric disorders, particularly, depression and anxiety are found at a higher frequency among MS patients (52). These conditions can affect a patient’s perception of relapse as well as her tolerance of symptoms, which can further blur the line between a real and a pseudo-relapse. Thus, a better assessment of the validity and severity of relapse would benefit these patients. In a recent study, depression has been found to be correlated with relapse severity (53). Depression can also be related to drug and alcohol abuse, which can further exacerbate motor and cognitive impairments during relapse (54). Furthermore, screening for depression both prior to and after treatment is critical when administering high dose corticosteroids for relapse, as these drugs have been associated with increased risk of attempted suicide (55). Alleviating depressive symptoms through a combination of antidepressants (56) and cognitive behavioral therapy (57) can help with symptom relief in some patients. Since antidepressants can affect the immune system and adverse side effects can lead to non-compliance, depression treatments need to be individually tailored based on tolerance, DMT, and social support system (58). As cognitive behavioral therapy can also improve sleep quality (59), it may be the preferred option for those affected by both mood and sleep disorders.

Physical comorbidities and lifestyle factors can also affect relapse. Diet is a modifiable lifestyle factor, which can impact the severity of disability and relapses in MS patients (60). A poor diet is also associated with the development of metabolic disorders such as obesity and diabetes. Obesity, particularly during adolescence, has emerged as a risk factor for developing MS (61), and obese MS patients experience higher rates of comorbidities and greater disability (62). This is believed to stem from the pro-inflammatory effects of adipokines released from adipose tissue (63). Central obesity, as defined by increased waist circumference, is often indicative of metabolic syndrome, and is suggested to be a more potent risk factor than body mass index alone (64). Consequently, efforts to promote a healthy weight and positively impact metabolic function can be beneficial for MS patients. Indeed, a recent study showed that treatment of diabetes, irrespective of MS treatment condition, could improve both metabolic condition and MS relapse rate (65). Therefore, the treatment of a comorbidity can sometimes positively impact relapse rates and improve patient outcomes. In contrast, some MS treatments have been implicated in the exacerbation of comorbidities (66), which could in turn negatively impact the prognosis of the patient in the long run. For example, corticosteroid treatment can cause hyperglycemia and exacerbate comorbid diabetes. However, if MS symptoms continue to worsen, it may be necessary to switch to a more potent DMT (67). Since patients with comorbidities are generally excluded from clinical trials, more work needs to be done at assessing the best treatment combinations for patients with comorbidities.

## Sex Hormones and MS Relapse

Unlike many other physiological states, which can exacerbate MS, pregnancy usually results in temporary relief from RRMS activity. A meta-analysis concluded that pregnancy is associated with a significant decrease, while the postpartum period is associated with an increase in MS activity (68). Levels of sex steroids including estrogen and progesterone fluctuate naturally during a woman’s menstrual cycle, and some patients with RRMS report increases in relapse rate or severity during the luteal phase, when the ratio of estrogen to progesterone is lowest (69). Women with RRMS have also been found to have lower levels of estrogen during the follicular phase, as compared to healthy controls (70). Similarly, when taking hormone-based oral contraceptives, women report increased symptoms during the week when hormones are absent from the pill (71). Furthermore, the onset of menopause is associated with the worsening of MS (72). Interestingly, a similar trend of symptomatic relief during pregnancy and increased relapse severity during menopause and postpartum periods has been noted for RA patients (73), suggesting a common role for estrogen in these two inflammatory autoimmune disorders.

A growing number of candidates have emerged as potential complementary or alternative treatments for MS (74), which are used in conjunction with traditional DMTs. Some guidelines have been proposed to inform patients and practitioners about the likely safety and efficacy of these adjuncts (75). However, it will ultimately be necessary to clinically validate these complementary treatments with a variety of DMTs, since the mechanism of the primary therapy could influence the function or efficacy of the adjunct. Recently, the therapeutic value of female sex hormones in RRMS on relapse has been investigated in phase 2 clinical trials. These phase 2 trials have demonstrated a therapeutic benefit for estrogen when used in combination with the DMTs glatiramer acetate (76) and interferon-β (77) and warrant further phase 3 studies. The role of progesterone is less clear, as the study investigating its use was terminated prematurely (78). In the context of RA, however, hormone replacement therapy is generally not advised for postmenopausal women due to the small degree of benefit in relation to the risk of cardiovascular side effects (79, 80). The use of oral contraceptives has generally been found to be neutral (81) or beneficial (82) for RRMS, but the outcome may be related to progestin content (83). Since birth control pills vary in terms of potency and composition of sex steroids, they may also differentially affect relapse rate and MS progression.

Estrogen is involved in anti-inflammatory processes and can affect the release of cytokines and chemokines from immune cells. Estrogen has also been shown to induce beneficial T regulatory cells in rodent EAE models and in humans (84). It has also been reported that in men with MS, serum levels of sex hormones and the level of estrogen receptors localized to the T regulatory cells are decreased (85), suggesting that the role of estrogen in MS is not restricted to women. While estrogen treatment is not a clinically viable treatment for men, testosterone is widely understood to be protective against autoimmune disorders, including MS. Indeed, decreased testosterone levels are risk factors for the development of MS in men (86). A small study recently reported a beneficial effect of testosterone therapy in reducing gray matter loss and associated cognitive decline (87), which may be related to the ability of androgens to act as re-myelination agents (88). Furthermore, the anti-inflammatory properties of both estrogens and androgens may converge on similar mechanisms toward the induction and expansion of T regulatory cells (89). Future research devoted to increasing our understanding of the underlying physiology for MS may help tailor the development of therapeutics, which benefit both genders.

## Author Contributions

EAM, AM, and YM-D contributed to the drafting and preparation of the manuscript and approved it in its final form. AM created the diagram and EAM and YM-D provided its details.

## Conflict of Interest Statement

EAM and AM have no conflicts of interest. No commercial funding was received to support this work. YM-D has served as a consultant and/or received grant support from: Acorda, Bayer Pharmaceutical, Biogen Idec, EMD Serono, Genzyme, Novartis, Questor, Chugai, and Teva Neuroscience.
